# Pacemaker Dependency in Patients Undergoing Implantation of Dual-chamber Devices After Alcohol Septum Ablation: A Retrospective Analysis

**DOI:** 10.1016/j.cjco.2026.06.021

**Published:** 2026-07-17

**Authors:** Sophia Schulze Lammers, Dennis Lawin, Annika Hoyer, Christoph Stellbrink, Thorsten Lawrenz

**Affiliations:** a Department of Cardiology and Intensive Care Medicine, Medical School and University Medical Center OWL, Public Hospital of Bielefeld, Bielefeld University, Bielefeld, Germany; b Biostatistics and Medical Biometry, Medical School OWL, Bielefeld University, Bielefeld, Germany

**Keywords:** alcohol septum ablation, atrioventricular block, hypertrophic cardiomyopathy, pacemaker

## Abstract

**Background:**

Patients with hypertrophic obstructive cardiomyopathy (HOCM) undergoing alcohol septal ablation (ASA) are at risk for high-degree atrioventricular (AV) block, often requiring permanent pacemaker (PPM) implantation. However, long-term pacemaker dependency in this population remains unclear.

**Methods:**

We retrospectively analyzed HOCM patients who underwent ASA and subsequent implantation of a cardiac implantable electronic device (CIED) due to procedure-related AV block between 2016 and 2023. CIED interrogation at ≥ 6 weeks postimplantation was used to assess ventricular pacing (VP). Pacemaker dependency was defined as > 1% VP. Associations with baseline characteristics were examined using logistic regression.

**Results:**

Of 464 patients undergoing ASA, 95 (20.5%) received a CIED, and 67 were available for follow-up. Only 30 patients (44.8%) showed > 1% VP; 9 (13.4%) were completely dependent (100% VP). In contrast, 31 patients (46.3%) had ≤ 0.1% VP. Persistent AV block was more common in patients with preinterventional left bundle branch block. Intraprocedural AV block showed a trend toward persistent pacemaker dependency.

**Conclusions:**

In this cohort, long-term pacemaker dependency after ASA was unexpectedly low. These findings suggest that AV conduction may recover in a significant proportion of patients, raising the question of whether some may safely avoid PPM implantation. Larger prospective studies are needed to guide device therapy decisions in this population. Our findings challenge the assumption of permanent AV block after ASA and suggest reconsidering routine early device implantation in selected patients.

## Résumé

**Contexte:**

Les patients atteints de cardiomyopathie hypertrophique obstructive (CHMO) subissant une ablation septale à l’alcool (ASA) présentent un risque de bloc auriculo-ventriculaire (AV) de haut grade, nécessitant souvent la pose d’un stimulateur cardiaque permanent (SCP). Cependant, la dépendance à long terme au stimulateur cardiaque chez cette population demeure mal définie.

**Méthodologie:**

Nous avons analysé rétrospectivement les patients atteints de CMHO ayant subi une ASA suivie de la pose d’un dispositif électronique cardiaque implantable (DECI) en raison d’un bloc AV lié à l’intervention, entre 2016 et 2023. L’interrogation du DECI, réalisée au moins 6 semaines après l’intervention a permis d’évaluer la stimulation ventriculaire (SV). La dépendance au stimulateur cardiaque a été définie comme une SV >1%. Une régression logistique a permis d’évaluer l’association entre les caractéristiques initiales et la dépendance au stimulateur.

**Résultats:**

Sur les 464 patients ayant subi une ASA, 95 (20,5 %) ont reçu un DECI, et 67 ont pu faire l’objet d’un suivi. Seuls 30 patients (44,8 %) présentaient une SV supérieure à 1 %; 9 (13,4 %) étaient totalement dépendants (100 % de SV). En revanche, 31 patients (46,3 %) présentaient une SV inférieure ou égale à 0,1 %. La persistance du bloc AV était plus fréquente chez les patients présentant un bloc de branche gauche avant l’intervention. Le bloc AV peropératoire montrait une tendance vers une dépendance persistante au stimulateur cardiaque.

**Conclusions:**

Dans cette cohorte, la dépendance à long terme au stimulateur cardiaque après une ASA s’est avérée étonnamment faible. Ces résultats suggèrent que la conduction AV pourrait se rétablir pour une proportion significative de patients, ce qui soulève la question de savoir si certains d’entre eux pourraient éviter, en toute sécurité, la pose d’un SCP. Des études prospectives à plus grande échelle sont nécessaires pour orienter les décisions thérapeutiques relatives aux dispositifs chez cette population. Nos résultats remettent en cause l’hypothèse d’un bloc AV permanent après une ASA et suggèrent de reconsidérer la pose systématique et précoce d’un dispositif chez certains patients sélectionnés.

The prevalence of hypertrophic cardiomyopathy (HCM) is estimated to be 1:300,^1^ with hypertrophic obstructive cardiomyopathy (HOCM) presenting in up to two thirds of patients with significant left ventricular outflow tract (LVOT) obstruction, defined as a systolic pressure gradient > 50 mm Hg between the left ventricle and ascending aorta.^2^^,^^3^ Patients with HOCM often experience dyspnea and angina pectoris on exertion. To improve these symptoms, septal reduction therapy by transcoronary injection of alcohol has been available since 1995.^4^ The results of alcohol septum ablation (ASA) are comparable to those of surgical myectomy in terms of LVOT gradient reduction, long-term survival, and sudden cardiac death rate.^5^, ^6^, ^7^ In the current guidelines of the **E**uropean **S**ociety of **C**ardiology (ESC), ASA is recommended as a treatment option for symptom improvement^3^ in drug-refractory HOCM patients. Today, ASA is the first option of septal reduction therapy in most countries due to its lower periprocedural morbidity and mortality.^6^ All forms of septal reduction therapies are associated with a risk of third-degree atrioventricular (AV) block requiring cardiac implantable electronic device (CIED) implantation. According to recent studies, the probability of a postinterventional AV block after ASA is approximately 14%-28%.^8^, ^9^, ^10^, ^11^ Because most HOCM patients undergo ASA at an early age, devices are frequently implanted for decades. This may be associated with an increased risk of long-term device complications, such as device malfunction. Especially in this scenario, the use of myosin inhibitors (eg, Mavacamten or Aficamten) in HOCM will soon be of special interest. However, it remains unclear whether the ASA-induced third-degree AV block persists in all patients or if AV conduction may recover after ASA in a subset of patients. Although the short-term recovery of AV-node conduction is already known and therefore several days are usually waited before a permanent pacemaker (PPM) is implanted, little is known about the long-term recoverability of the AV node after ASA. Therefore, our aim was to investigate the persistence of third-degree AV block in patients who experienced AV block during ASA and subsequently received a CIED, and the predictors for this complication.

## Methods

### Study population

We retrospectively included all patients who underwent ASA at our institution between 2016 and 2023 (n = 464) and received a postprocedural CIED due to AV block (n = 95). Exclusion criteria included pre-existing PPM or implantable cardioverter defibrillator (ICD) implantation, missing follow-up data (eg, unavailable CIED interrogation ≥ 6 weeks after implantation), and sequential AV pacing for symptomatic HCM (n = 25). Data collection followed the principles outlined in the Declaration of Helsinki (2013) and Good Clinical Practices. In addition, the study was approved by the ethics committee of the Ärztekammer Westfalen-Lippe (2024-112-f-S) and all patients signed an informed consent form prior to participation. The authors confirm that patient consent forms have been obtained for this article.

A total of 95 of 464 patients required postprocedural CIED for high-degree AV block and 67 of these patients had no exclusion criteria and were included in the study (Fig. 1). Baseline characteristics (age, sex, preprocedural electrocardiology [ECG] data), procedure details (amount of ethanol injected, number of branches with injected ethanol), and the percentage of ventricular pacing (VP) obtained from CIED measurements at least 6 weeks after ASA were obtained from medical records. Potential associations between baseline characteristics and the persistence of AV block were analyzed using logistic regression.

**Figure 1:**
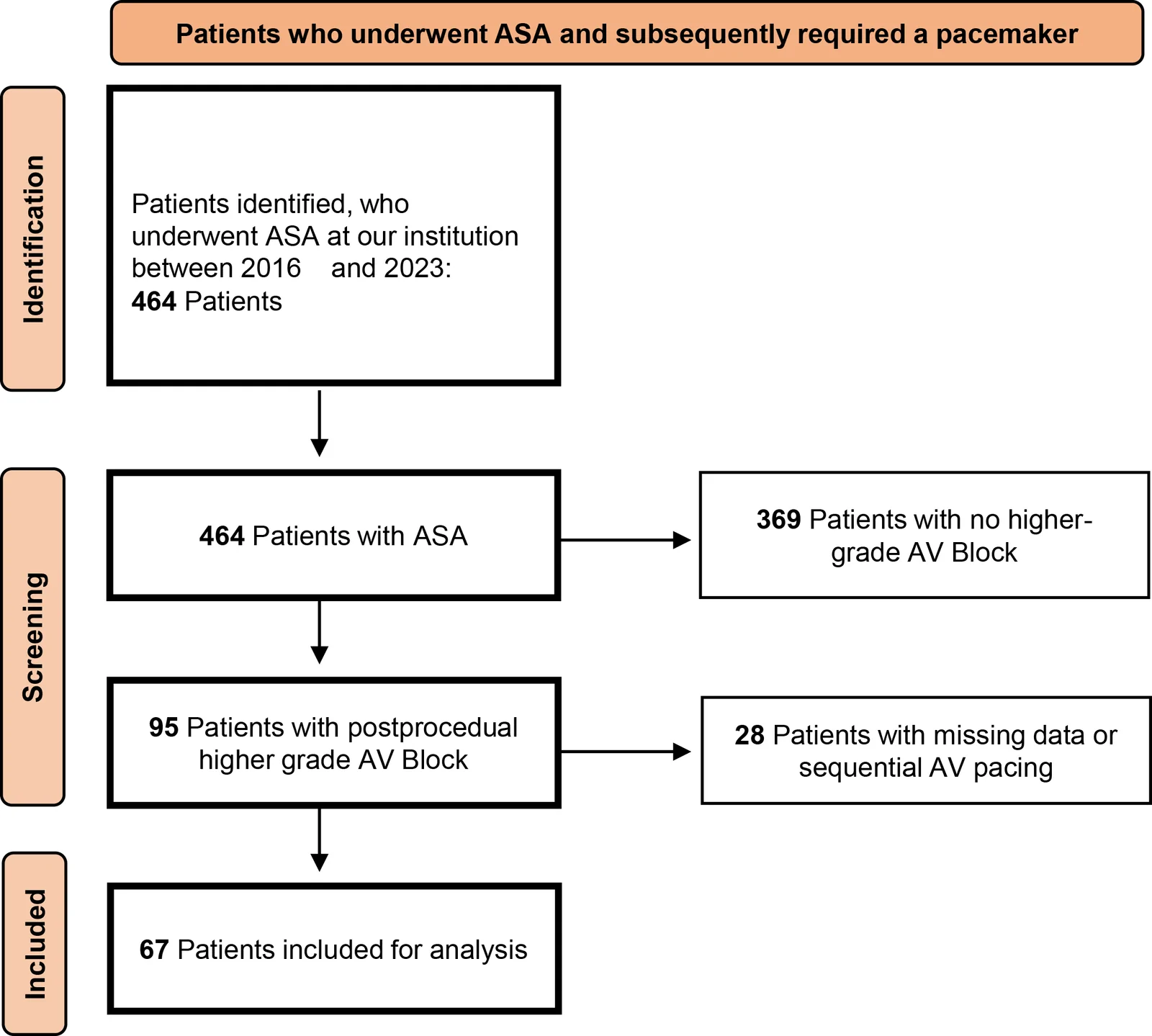
Study design. We identified 464 patients who underwent ASA during the study period. Patients who had previously received a permanent pacemaker (or implantable cardioverter defibrillator) and patients who did not receive a cardiac implantable electronic device (CIED) were excluded. No or incomplete follow-up data were available for 28 patients. Sixty-seven patients who received a CIED due to AV block were examined for pacemaker dependency as part of the long-term follow-up. ASA, alcohol septum ablation; AV, atrioventricular.

### Therapeutic procedure

Patients with symptomatic HOCM despite maximal medical therapy and a baseline or provoked maximal LVOT gradient of ≥ 50 mm Hg underwent ASA according to the current ESC guidelines.^3^ Details of the procedure, such as volume of alcohol injected and number of septal branches, were at the discretion of the operator.

All patients received a temporary pacemaker after the ASA. The device was left in place for at least 24 hours in all patients. In high-risk patients, temporary pacemaker backup was maintained for 4 days after ASA. High-risk status was defined as retrograde AV block after ASA in combination with at least 1 additional risk factor (age > 58 years, intraprocedural third-degree AV block, or postprocedural QRS duration > 120 ms).^8^^,^^12^ In addition, a telemetric rhythm monitoring for 7 days was performed at an intermediate care unit.

### Definition of ASA-induced third-degree AV block

A transient third-degree AV block was defined as a block of at least 10 seconds. A persistent third-degree AV block was defined as a duration of a block of at least 30 minutes.

We differentiated 4 groups of patients: The first group had intraprocedural transient total heart block, where third-degree AV block occurred for > 10 seconds during the ASA procedure and recovered completely during the intervention. The second group included patients with intraprocedural sustained total heart block, in which a third-degree AV block occurred during the ASA procedure and lasted for > 30 minutes.

The third group included those with postprocedural transient total heart block, defined as third-degree AV block lasting ≥ 10 seconds during telemetric monitoring 5-7 days after ASA, followed by spontaneous recovery. No CIED was implanted in cases of strictly intraprocedural transient third-degree AV block.

Finally, the fourth group of patients, with a postprocedural persistent total heart block, presented with third-degree AV block that occurred during the postprocedural telemetric monitoring after the ASA procedure that persisted for ≥ 30 minutes.

For safety reasons, CIED implantation was performed in patients with persistent or postprocedural transient total heart block. This strategy was chosen because late transient postprocedural AV block with syncope was observed several weeks after ASA in some cases.

In patients with recovered AV conduction, a sensed AV interval of 150 ms and a paced AV interval of 180 ms were programmed. AV optimization algorithms were not activated during the first 6 months after ASA to allow for conduction remodeling. Device manufacturers included Medtronic (90%), Abbott/St Jude Medical (5%), and Biotronik (5%).

Patients at high risk of sudden cardiac death were implanted with a dual-chamber ICD in cases of persistent or postprocedural transient total heart block, in accordance with the guidelines of the ESC.^3^

### Outcomes

All patients underwent their first pacemaker check-up 4 weeks after implantation. Further device follow-up records were retrieved for all patients between 6 weeks and 32 months after implantation at our institution, or the data were obtained from the patients' cardiologists.

Because pacemaker check-ups analyze the period from the last analysis to the date of the check-up, the first 4-week period postimplantation was not included in the final evaluation for any patient. The case of pacemaker dependence immediately after implantation and the restoration of AV-node conduction during the subsequent follow-up examination can therefore be ruled out.

In this study we examined the most recent available PPM survey, and it was determined that pacemaker dependency was present if VP exceeded 1%. VP fractions between 0% and 1% were defined as no pacemaker dependence.

A threshold value of > 1% VP has already been used in several studies as a practical cutoff for the analysis of right ventricular (RV) pacing---for example, both Hartrampf et al. and Thibault et al. considered > 1% RV pacing to be an indication of non-negligible pacemaker activity.^13^^,^^14^ Even if this is not a formal definition of pacemaker dependence in the physiologic sense, the threshold value allows for an objective and comparable grouping.

### Statistical analysis

We report mean (standard deviation [SD]) or median [quartile 1 to quartile 3] for continuous variables and number of observations (proportion) for categorical variables. Logistic regression was performed to assess potential associations between persistent AV block and baseline characteristics (age, sex, initial ECG pathology, indication for PPM [persistent or paroxysmal AV block], time of occurrence of the AV block [intra- or postprocedural], or amount of applied ethanol). Odds ratios (ORs) are given with their 95% confidence intervals (CIs) and *P* values. All analyses were performed using SAS version 9.4 (SAS Institute, Cary, NC).

## Results

In our retrospective analysis, we identified 464 patients who underwent ASA due to symptomatic HOCM at our institution between 2016 and 2023. In 95 of these, ASA-induced third-degree AV block required implantation of a dual-chamber PPM or ICD, which corresponds to a rate of 20.5%. Of these 95 patients, 67 (70.5%) had a complete follow-up (FU) of ≥ 6 weeks after implantation and could therefore be included in the study.

The median age of included patients was 60 (53.00-69.00) years and 55.22% of the patients (n = 37) were male; 41.79% of the patients (n = 28) received an ICD due to an elevated HCM risk sudden cardiac death score (≥ 4%/5 years). Of the 67 patients, 61.19% (n = 41) had no ECG changes before ASA, 20.9% (n = 14) of them showed a left bundle branch block (LBBB), 14.93% (n = 10) a right bundle branch block (RBBB), and in 2.98% (n = 2) a left anterior fascicular block was present.

In 38 patients (56.72%) an intraprocedural third-degree AV block occurred. Of these 38 patients, 29 (43.28%) had intraprocedural third-degree AV block that persisted postprocedurally, and 9 patients (13.44%) had temporary intraprocedural third-degree AV block that recurred after the procedure.

Postprocedural third-degree AV block was observed in the remaining 29 patients (43.28%), with at least 1 episode of transient total heart block occurring in 26 patients (38.8%) and persistent total heart block in 3 patients (4.48%).

The mean amount of ethanol injected was 1.90 mL (SD 0.66), with 20 patients (29.85%) receiving > 2 mL of ethanol. The mean number of intervened branches was 1.07 (SD 0.26). An overview of the baseline characteristics is shown in Table 1.

**Table 1:** Baseline characteristics (N = 67)

Variable	Value
Women, n (%)	30 (44.78%)
Men, n (%)	37 (55.22%)
Age, years, median (Q1-Q3)	60.00 (53-69)
Prior LBBB, n (%)	14 (20.90%)
Prior RBBB, n (%)	10 (14.93%)
Prior LAFB, n (%)	2 (2.98%)
Intraprocedural third-degree AV block (total), n (%)	38 (56.72%)
Intraprocedural transient third-degree AV block, n (%)	9 (13.44%)
Intraprocedural persistent third-degree AV block, n (%)	29 (43.28%)
Postprocedural third-degree AV block (total), n (%)	29 (43.28%)
Postprocedural transient third-degree AV block, n (%)	26 (38.8%)
Postprocedural persistent third-degree AV block, n (%)	3 (4.48%)
Total transient third-degree AV block, n (%)	36 (53.73%)
Total persistent third-degree AV block, n (%)	31 (46.27%)
Ethanol injected, mL, mean (SD)	1.90 (0.66)
> 2 mL ethanol injected, n (%)	20 (29.85%)
ICD implanted, n (%)	28 (41.79%)

AV, atrioventricular; ICD, implantable cardioverter defibrillator; LAFB, left anterior fascicular block; LBBB, left bundle branch block; Q, quartile; RBBB, right bundle branch block; SD, standard deviation.

The median FU was 29 months after ASA. After analyzing the CIED interrogation data, it was found that 30 of 67 patients (44.78%) still required VP, defined as VP > 1%. Nine of these 30 patients (13.43%) were permanently dependent on VP; that is, they had 100% VP and inhibition of the device showed total heart block in all these patients. In contrast, 37 patients (55.22%) had ≤ 1% VP and 31 (46.27%) had even ≤ 0.1% VP. Seven patients (10.45%) had VPs between 1.1% and 29.9%, 2 patients (2.99%) had VPs between 30% and 95%, and 12 patients (17.91%) had VPs between 95% and 99.9%. The distribution of VP rates is shown in Figure 2.

**Figure 2:**
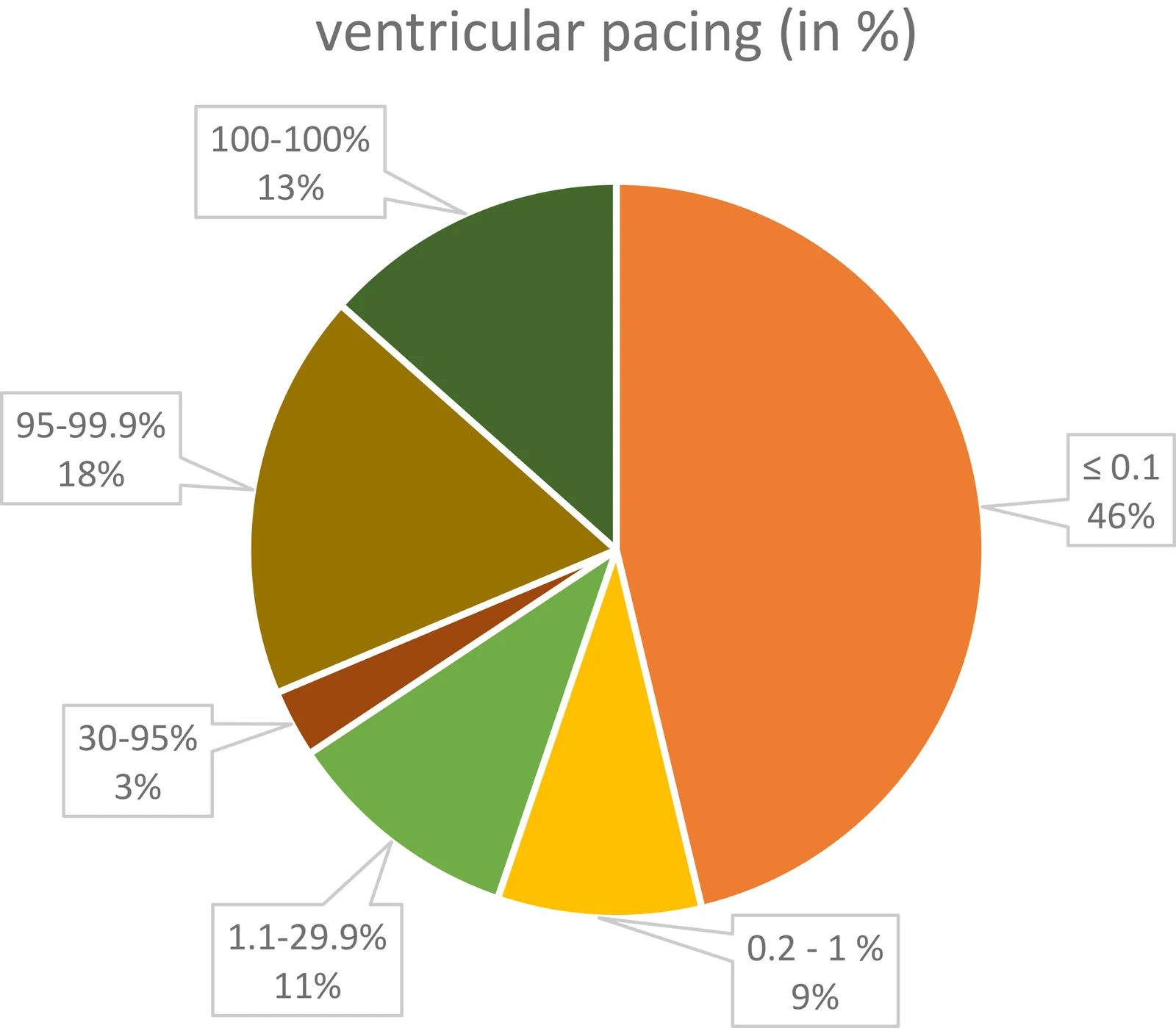
Distribution of patients according to their ventricular pacing rates at follow-up.

Women were less likely to have a persistent third-degree AV block than men at FU, but the difference was not statistically significant (OR = 0.46 [0.14-1.57], *P* = 0.22). In contrast, there was an association between pre-existing ECG abnormalities and pacing rate during FU: Patients with LBBB showed a 38% greater chance of persistent third-degree AV block than patients without initial ECG pathology (OR = 1.38 [0.33-5.76], *P* = 0.65). Moreover, in patients with intraprocedural total heart block, the chance for a recurrence of third-degree AV block was higher during FU vs patients with postprocedural total heart block (OR = 1.93 [0.35-10.74], *P* = 0.45). This was particularly evident in intraprocedural persistent third-degree AV blocks vs intraprocedural transient blocks (OR = 1.52 [0.3-7.6], *P* = 0.61). In device-dependent patients, the likelihood of a persistent AV block was higher with an increasing amount of ethanol injected during ASA (OR = 1.54 [0.56-4.21], *P* = 0.39). Looking at the age of the patients and the persistence of third-degree AV block, the chance of persistence increased with older age (OR = 1.069 [1.01-1.13], *P* = 0.018).

Table 2 shows the baseline characteristics according to continuing device dependence.

**Table 2:** Baseline characteristics according to persistence of third-degree AV block

	Overall (N = 67)	Pacemaker dependency at FU (VP > 1%) (n = 30)	No pacemaker dependency at FU (VP ≤ 1%) (n = 37)
Women, n (%)	30 (44.8%)	13 (43.3%)	17 (56.7%)
Men, n (%)	37 (55.2%)	17 (45.9%)	20 (54.1%)
Age, years, median (Q1-Q3)	59.6 (53-68)	63.00 (57.00-74.00)	55.00 (51.50-65.50)
Pre-existing LBBB, n (%)	14 (20.9%)	9 (64.3%)	5 (35.7%)
Pre-existing RBBB, n (%)	10 (14.9%)	4 (40%)	6 (60%)
Pre-existing LAFB, n (%)	2 (2.9%)	1 (33.3%)	2 (66.7%)
Pre-existing first-degree AV block, n (%)	3 (4.5%)	1 (33.3%)	2 (66.7%)
Intraprocedural third-degree AV block, n (%)	38 (56.7%)	21 (55.3%)	17 (44.7%)
Intraprocedural transient third-degree AV block, n (%)	9 (13.4%)	4 (44.4%)	5 (55.6%)
Intraprocedural persistent third-degree AV block, n (%)	29 (43.3%)	17 (58.6%)	12 (41.4%)
Postprocedural third-degree AV block, n (%)	32 (47.8%)	9 (28.1%)	23 (71.9%)
Postprocedural transient third-degree AV block, n (%)	28 (41.8%)	8 (28.6%)	20 (71.4%)
Postprocedural persistent AV block, n (%)	4 (5.9%)	1 (25%)	3 (75%)
Transient third-degree AV block, n (%)	35 (52.2%)	12 (32%)	26 (68%)
Persistent third-degree AV block, n (%)	32 (47.8%)	18 (56.3%)	14 (43.75%)
Ethanol injected, mL, mean (SD)	1.87 (0.64)	1.88 (0.61)	1.90 (0.68)
> 2 mL ethanol injected, n (%)	20 (29.9%)	8 (40%)	12 (60%)
ICD implanted, n (%)	28 (41.8%)	10 (35.7%)	18 (64.3%)

AV, atrioventricular; ICD, implantable cardioverter defibrillator; LAFB, left anterior fascicular block; LBBB, left bundle branch block; Q, quartile; RBBB, right bundle branch block; SD, standard deviation; VP, ventricular pacing.

One result of our study is that pacemaker dependency in the first weeks after ASA could be divided into 2 groups: those predominantly receiving pacemaker stimulation and those receiving hardly any pacemaker stimulation (Fig. 3).

**Figure 3:**
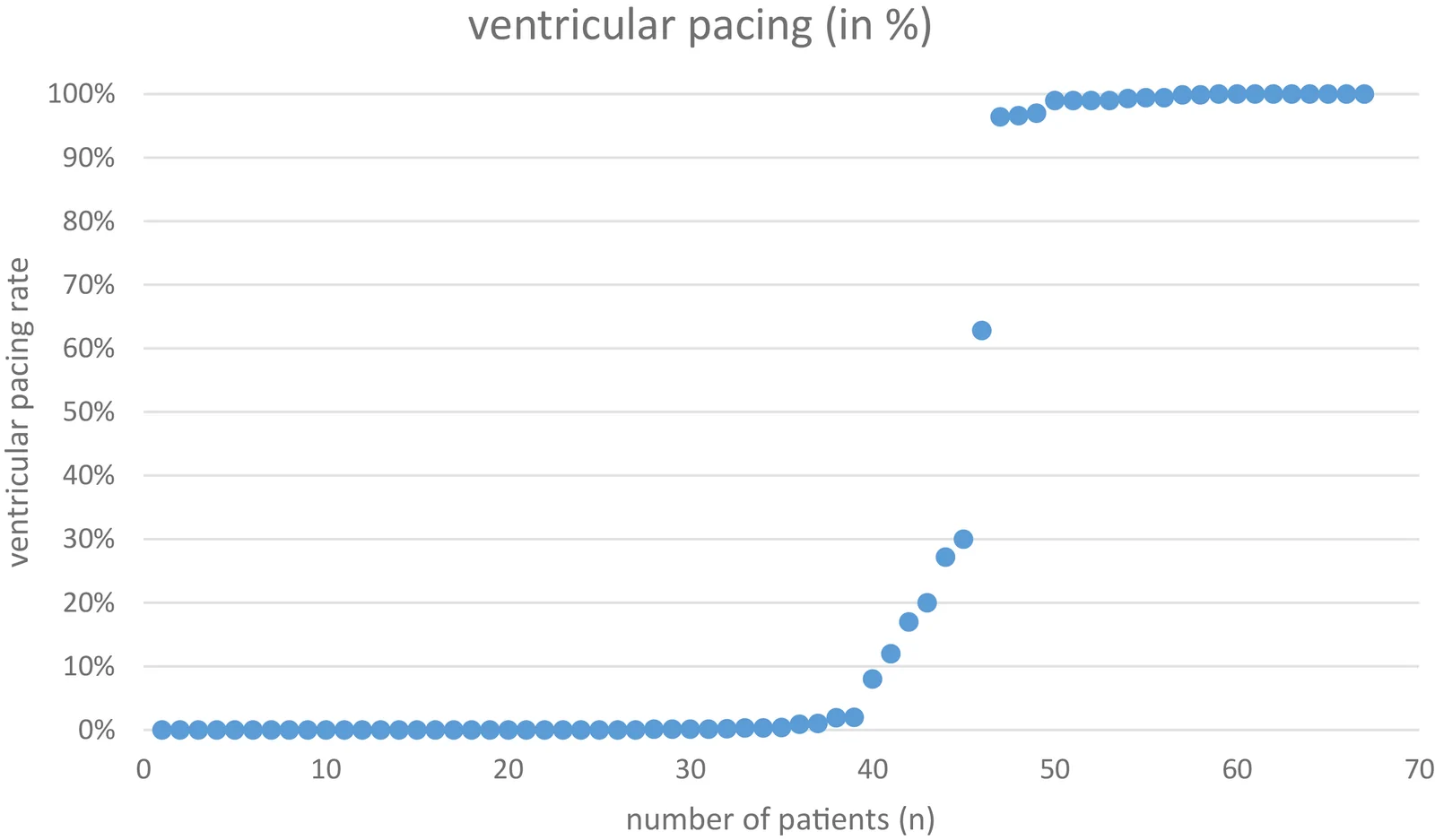
Patient distribution based on ventricular pacing rate. Each blue dot marks the ventricular pacing rate of a patient. The x axis shows patient number (n) and the y axis shows the ventricular pacing rate.

## Discussion

The long-term results of ASA treatment have been shown to be successful in previous studies (eg, Veselka et al.^12^). However, a relevant potential risk of septal reduction therapy is PPM dependency. Although the short-term recoverability of the AV node has already been well studied, there are only a few studies on the regenerative capacity after several years.^11^^,^^12^^,^^15^ Interestingly, the results of our study show a high recoverability of the AV node: < 50% of patients are permanently pacemaker-dependent. At FU, it was found that AV-node conduction often recovered after just a few weeks. This raises the question of whether pacemaker implantation is permanently necessary after ASA.

However, for safety reasons it is mandatory to identify patients who require pacemaker stimulation very carefully and it is unclear at which pacing threshold this is safe for patients without a pacemaker back-up. Our study has shown that patients with an intraprocedural persisting third-degree AV block had the highest risk for PPM dependency during FU. Furthermore, transient AV block appears to recover better than persistent AV block. Patients with an increased amount of injected ethanol of > 2.0 mL also showed a higher rate of pacemaker dependency in FU.

The risk parameters for the development of an intra- or postprocedural AV block have been known for some time; that is, female sex, first-degree AV block, presence of LBBB, and injection of ethanol in more than 1 septal artery have been shown to be associated with a higher risk of AV block.^15^^,^^16^ More recent studies also depict that older age (defined as age > 50 years) is associated with a higher risk of CIED treatment.^17^ RBBB, on the other hand, no longer appears to be associated with CIED treatment.^18^ We have now shown similar results with regard to the persistence of the AV block: If an AV block occurs periprocedurally, patients with a pre-existing LBBB have an increased chance of remaining permanently pacemaker-dependent. In contrast, with an RBBB, there is no evidence of an association with persistent AV block. In addition, it has been shown that the chance of permanent AV blockage increases with advanced age (*P* = 0.018). Thus, regenerability of the AV node appears to be age dependent. It appears likely that there is either a complete recovery of the AV block or not.

To date, there have been hardly any trials on the time course of pacemaker dependency during midterm FU. For example, a study that monitored 25 patients after ASA with CIED showed 64% pacemaker dependency.^19^ However, PPM dependency was defined as a threshold value of 5% VP in that study. Even if we transfer these results onto our study, the result is a lower value of 44.8% for AV block persistence. Currently, pacemaker devices are unable to store the internal electrocardiogram prior to or during the pacing period. Therefore, we cannot define a safe threshold at which pacemaker back-up may be no longer needed after ASA. Even 0.1% VP may result in syncope if caused by a short total heart block. Consequently, even patients with minimal pacing fractions may still require PM support at critical moments. It will therefore be difficult to define a threshold value at which the pacemaker is not needed and explantation can take place. Still, programming errors such as unnecessarily high ventricular stimulation must be ruled out. On the other hand, device-related complications must be considered.

Our study has shown that the potential for recovery of AV-node conduction is very high, especially in transient postprocedural total heart block. Therefore, indications for PPM implantation in this scenario should be carefully assessed. In transient postprocedural total heart block, it appears reasonable to extend the duration of telemetric monitoring after a watchful waiting strategy. The implantation of an unnecessary permanent device could be prevented in patients with full recovery of AV-node conduction for several days.

Further studies will be needed to identify the exact time interval of telemetric monitoring that allows for safe prediction in patients with postprocedural transient AV block. This is of utmost importance, especially in view the different health care systems in different countries, as prolongation of telemetric monitoring greatly enhances the costs of the procedure. On the other hand, the side effects and the FU costs of an unnecessarily implanted device must also be considered. Taken together, however, our data support the strategy of early pacemaker implantation in patients with persisting AV block after ASA and a watchful waiting strategy in patients with transient postprocedural total heart block.

### Limitations

The retrospective design and the relatively short FU period represent limitations to our study.

## Conclusions

Our findings reveal a heretofore unexpected potential of recovery of AV-node conduction after ASA, especially in postprocedural transient third-degree AV block. A watchful waiting strategy appears reasonable in these patients before direct implantation of a device in this subgroup of patients.

## Data Availability

The authors confirm that all data generated or analyzed during this study are included in this published article. For sensitive reasons, the data supporting the study's findings are not publicly available.

## Ethics Statement

The research reported in this paper has adhered to the principles of the Declaration of Helsinki. The study was approved by the ethics committee of the Ärztekammer Westfalen-Lippe (2024-112-f-S).

## Patient Consent

The authors confirm that patient consent is not applicable to this article. This is a retrospective case report using de-identified data; therefore, the institutional review board did not require consent from the patients.

## Funding Sources

The authors have no funding sources to declare.

## Disclosures

The authors have no conflicts of interest to disclose.

## References

[1] Husser D., Ueberham L., Jacob J. (2018). Prevalence of clinically apparent hypertrophic cardiomyopathy in Germany---an analysis of over 5 million patients. PLoS One.

[2] Kuhn H., Mercier J., Köhler E. (1983). Differential diagnosis of hypertrophic cardiomyopathies: typical (subaortic) hypertrophic obstructive cardiomyopathy, atypical (mid-ventricular) hypertrophic obstructive cardiomyopathy and hypertrophic non-obstructive cardiomyopathy. Eur Heart J.

[3] Sigwart U. (1995). Non-surgical myocardial reduction for hypertrophic obstructive cardiomyopathy. Lancet.

[4] Ralph-Edwards A., Woo A., McCrindle B.W. (2005). Hypertrophic obstructive cardiomyopathy: comparison of outcomes after myectomy or alcohol ablation adjusted by propensity score. J Thorac Cardiovasc Surg.

[5] Kim L.K., Swaminathan R.V., Looser P. (2016). Hospital volume outcomes after septal myectomy and alcohol septal ablation for treatment of obstructive hypertrophic cardiomyopathy: US Nationwide Inpatient Database, 2003-2011. JAMA Cardiol.

[6] Arbelo E., Protonotarios A., Gimeno J.R. (2023). 2023 ESC Guidelines for the management of cardiomyopathies. Eur Heart J.

[7] Fifer M.A., Sigwart U. (2011). Controversies in cardiovascular medicine. Hypertrophic obstructive cardiomyopathy: alcohol septal ablation. Eur Heart J.

[8] Lawrenz T., Lieder F., Bartelsmeier M. (2007). Predictors of complete heart block after transcoronary ablation of septal hypertrophy: results of a prospective electrophysiological investigation in 172 patients with hypertrophic obstructive cardiomyopathy. J Am Coll Cardiol.

[9] Grazina A., Cardoso I., Fiarresga A. (2024). Permanent pacemaker implantation after alcoholic septal ablation induced complete heart block: long-term impact. Rev Port Cardiol.

[10] Jensen M.K., Faber L., Liebregts M. (2019). Effect of impaired cardiac conduction after alcohol septal ablation on clinical outcomes: insights from the Euro-ASA registry. Eur Heart J Qual Care Clin Outcomes.

[11] Veselka J., Liebregts M., Cooper R. (2022). Outcomes of patients with hypertrophic obstructive cardiomyopathy and pacemaker implanted after alcohol septal ablation. JACC Cardiovasc Interv.

[12] Veselka J., Lawrenz T., Stellbrink C. (2013). Low incidence of procedure-related major adverse cardiac events after alcohol septal ablation for symptomatic hypertrophic obstructive cardiomyopathy. Can J Cardiol.

[13] Hartrampf B., Jochheim D., Steffen J. (2021). Permanent pacemaker dependency in patients with new left bundle branch block and new first degree atrioventricular block after transcatheter aortic valve implantation. Sci Rep.

[14] Thibault B., Ducharme A., Baranchuk A. (2015). Very low ventricular pacing rates can be achieved safely in a heterogeneous pacemaker population and provide clinical benefits: the CANadian Multi-Centre Randomised Study-Spontaneous AtrioVEntricular Conduction pReservation (CAN-SAVE R) Trial. J Am Heart Assoc.

[15] Veselka J., Krejčí J., Tomašov P., Zemánek D. (2014). Long-term survival after alcohol septal ablation for hypertrophic obstructive cardiomyopathy: a comparison with general population. Eur Heart J.

[16] Chang S.M., Nagueh S.F., Spencer W.H., Lakkis N.M. (2003). Complete heart block: determinants and clinical impact in patients with hypertrophic obstructive cardiomyopathy undergoing nonsurgical septal reduction therapy. J Am Coll Cardiol.

[17] Karimianpour A., Heizer J., Leaphart D. (2021). Predicting complete heart block after alcohol septal ablation for hypertrophic cardiomyopathy using a risk stratification model and clinical tool. Cathet Cardiovasc Interv.

[18] Lawin D., Lawrenz T., Radke K., Wolff A., Stellbrink C. (2022). Alcohol-induced right bundle branch block is associated with a benign outcome in HOCM after alcohol septum ablation (ASA). Clin Res Cardiol.

[19] Bode W.D., Bode M.F., Zhao M. (2023). Predictors of cardiovascular implantable electronic device dependence at long-term follow-up after alcohol septal ablation in hypertrophic cardiomyopathy patients. J Interv Card Electrophysiol.

